# Cost-effectiveness of MRI targeted biopsy strategies for diagnosing prostate cancer in Singapore

**DOI:** 10.1186/s12913-021-06916-0

**Published:** 2021-09-03

**Authors:** Li-Jen Cheng, Swee Sung Soon, Teck Wei Tan, Cher Heng Tan, Terence Sey Kiat Lim, Kae Jack Tay, Wei Tim Loke, Bertrand Ang, Edmund Chiong, Kwong Ng

**Affiliations:** 1 Agency for Care Effectiveness, Ministry of Health, Singapore, 16 College Road, Singapore, 169854 Singapore; 2 Department of Urology, Tan Tock Seng Hospital, Singapore, Singapore; 3 Department of Diagnostic Radiology, Tan Tock Seng Hospital, Singapore, Singapore; 4 Lee Kong Chian School of Medicine, Nanyang Technological University, Singapore, Singapore; 5 Department of Urology, Changi General Hospital, Singapore, Singapore; 6 Department of Urology, Singapore General Hospital, Singapore, Singapore; 7 Urology Service, Ng Teng Fong General Hospital, Singapore, Singapore; 8 Department of Diagnostic Imaging, National University Hospital, Singapore, Singapore; 9 Department of Urology, National University Hospital, Singapore, Singapore; 10 Department of Surgery, Yong Loo Lin School of Medicine, National University of Singapore, Singapore, Singapore

## Abstract

**Background:**

To evaluate the cost-effectiveness of six diagnostic strategies involving magnetic resonance imaging (MRI) targeted biopsy for diagnosing prostate cancer in initial and repeat biopsy settings from the Singapore healthcare system perspective.

**Methods:**

A combined decision tree and Markov model was developed. The starting model population was men with mean age of 65 years referred for a first prostate biopsy due to clinical suspicion of prostate cancer. The six diagnostic strategies were selected for their relevance to local clinical practice. They comprised MRI targeted biopsy following a positive pre-biopsy multiparametric MRI (mpMRI) [Prostate Imaging – Reporting and Data System (PI-RADS) score ≥ 3], systematic biopsy, or saturation biopsy employed in different testing combinations and sequences. Deterministic base case analyses with sensitivity analyses were performed using costs from the healthcare system perspective and quality-adjusted life years (QALY) gained as the outcome measure to yield incremental cost-effectiveness ratios (ICERs).

**Results:**

Deterministic base case analyses showed that Strategy 1 (MRI targeted biopsy alone), Strategy 2 (MRI targeted biopsy ➔ systematic biopsy), and Strategy 4 (MRI targeted biopsy ➔ systematic biopsy ➔ saturation biopsy) were cost-effective options at a willingness-to-pay (WTP) threshold of US$20,000, with ICERs ranging from US$18,975 to US$19,458. Strategies involving MRI targeted biopsy in the repeat biopsy setting were dominated. Sensitivity analyses found the ICERs were affected mostly by changes to the annual discounting rate and prevalence of prostate cancer in men referred for first biopsy, ranging between US$15,755 to US$23,022. Probabilistic sensitivity analyses confirmed Strategy 1 to be the least costly, and Strategies 2 and 4 being the preferred strategies when WTP thresholds were US$20,000 and US$30,000, respectively.

**Limitations and conclusions:**

This study found MRI targeted biopsy to be cost-effective in diagnosing prostate cancer in the biopsy-naïve setting in Singapore.

**Supplementary Information:**

The online version contains supplementary material available at 10.1186/s12913-021-06916-0.

## Introduction

Prostate cancer is the second most common cancer diagnosed and the fifth most fatal cancer amongst men globally [1]. In Singapore, prostate cancer is the third most common cancer in men, accounting for 14.1% of cancers diagnosed and 5.8% of total cancer deaths in men from 2013 to 2017 [2]. The discordant incidence and mortality reflect prostate cancer’s indolent growth and low fatality especially when diagnosed without metastasis [3, 4], and potential overdiagnosis of clinically insignificant cancer partly contributed by the limitations of prostate-specific antigen (PSA) testing [5, 6]. Currently, there is no population-wide screening recommended for the early detection of prostate cancer in Singapore. Individual men aged 50 to 70 years with life expectancy exceeding 10 years may be offered PSA testing after discussing its potential benefits and harms [7].

Distinguishing clinically significant prostate cancer from clinically insignificant ones is central to the management of prostate cancer. While the definition of clinical significance continues to evolve [8], the underpinning approach is accurate detection and characterization of clinically significant cancer to improve morbidity and mortality while minimizing adverse effects of unnecessary treatments. Limiting treatment of clinically insignificant cancers that do not threaten life expectancy can reduce overdiagnosis and overtreatment [9].

Contemporary non-targeted transrectal or transperineal prostate biopsies rely on real-time ultrasound guidance. Despite good imaging of the prostate gland boundaries and its adjacent organ structures, ultrasound cannot distinguish malignant lesions from benign ones [10]. Ultrasound alone is also insufficient to target specific lesions as about 40% are isoechoic [11]. The inability to target specific lesions can lead to sampling errors and inaccurate risk stratification which can affect subsequent clinical management decisions. Targeted biopsy techniques can potentially circumvent these limitations. Accumulating evidence supports the use of prebiopsy multiparametric magnetic resonance imaging (mpMRI) followed by magnetic resonance imaging (MRI) targeted biopsy as they detect more high-grade cancers with fewer biopsy cores, while reducing detection of low-grade clinically insignificant cancers [12–15]. To assess the value of such tests, cost-effectiveness studies can be conducted to simulate costs and effects of a new testing strategy and subsequent treatment options compared to existing strategies. Most published cost-effectiveness studies focus on a single biopsy protocol which do not reflect the use of MRI targeted biopsy in real life when multiple diagnostic strategies are used. A comprehensive comparison of all clinically relevant diagnostic strategies involving MRI targeted biopsy positioned in various diagnostic sequences can shed light on resource allocation in prostate cancer diagnosis in the initial and repeat biopsy settings. This is particularly pertinent as mpMRI and MRI targeted biopsy are becoming the standard of care in diagnosing prostate cancer [16, 17].

This study aims to evaluate the cost-effectiveness of six diagnostic strategies involving MRI targeted biopsy for diagnosing prostate cancer in initial and repeat biopsy settings from the Singapore healthcare system perspective.

## Methods

### Patient population

The starting model population was men aged 65 years clinically suspected of having localized prostate cancer based on elevated serum PSA above 4 ng/ml, abnormal digital rectal examination (DRE), or both, and referred for a first prostate biopsy. The starting age of 65 years was used as it corresponded with the estimated age recording a marked increase in age-specific incidence rate of prostate cancer in Singapore [18]. Men presenting de novo with metastases due to prostate malignancy were excluded as they almost never require MRI targeted biopsy for initial prostate cancer diagnosis due to the presence of locally advanced cancer in addition to their metastases.

### Diagnostic strategies

Table 1 shows six diagnostic strategies relevant to the local practice that were evaluated in the model. They involved MRI targeted biopsy, systematic biopsy, and saturation biopsy, employed in different testing combinations and sequences. In Singapore’s context, MRI targeted biopsy refers to MRI-ultrasound (US) fusion targeted biopsy combined with systematic biopsy as a combined technique has been shown to improve detection of clinically significant cancer [19–22]. Locally, a 12-core systematic biopsy is the more common systematic biopsy performed. This definition of MRI targeted biopsy is consistent with a recent Cochrane review [23].

**Table 1:** Diagnostic strategies evaluated in the model

Strategy	Diagnostic pathway
Strategy 1	MRI targeted biopsy
Strategy 2	MRI targeted biopsy ➔ Systematic biopsy
Strategy 3	MRI targeted biopsy ➔ Saturation biopsy
Strategy 4	MRI targeted biopsy ➔ Systematic biopsy ➔ Saturation biopsy
Strategy 5	Systematic biopsy ➔ MRI targeted biopsy
Strategy 6	Systematic biopsy ➔ MRI targeted biopsy ➔ Saturation biopsy

***Abbreviation***: *MRI* magnetic resonance imaging

**Note**:

1. MRI targeted biopsy refers to the administration of MRI targeted biopsy combined with systematic biopsy following a positive mpMRI.

2. Arrow (➔) refers to next sequence of diagnostic test following a negative biopsy result.

A positive prebiopsy mpMRI with a Prostate Imaging – Reporting and Data System (PI-RADS) score of 3 to 5 was subjected to further testing by MRI targeted biopsy. In the initial biopsy setting, patients with negative mpMRI did not proceed to biopsy; in repeat biopsy settings, patients with negative mpMRI received systematic biopsy alone if there was persistent clinical suspicion. Although saturation biopsy is also a form of systematic biopsy, it typically involves extracting 20 or more cores [24]. Saturation biopsy was assumed to be template prostate mapping biopsy using a 5 mm sampling frame [25]. All prostate biopsies can be performed transrectally or transperineally with local or general anesthesia, and are associated with bleeding, infection, and urinary retention risks [26, 27]. The model assumed that patients received a maximum of three biopsies for initial diagnosis of prostate cancer if clinical suspicion remained.

### Model structure and key specifications

**Fig. 1:**
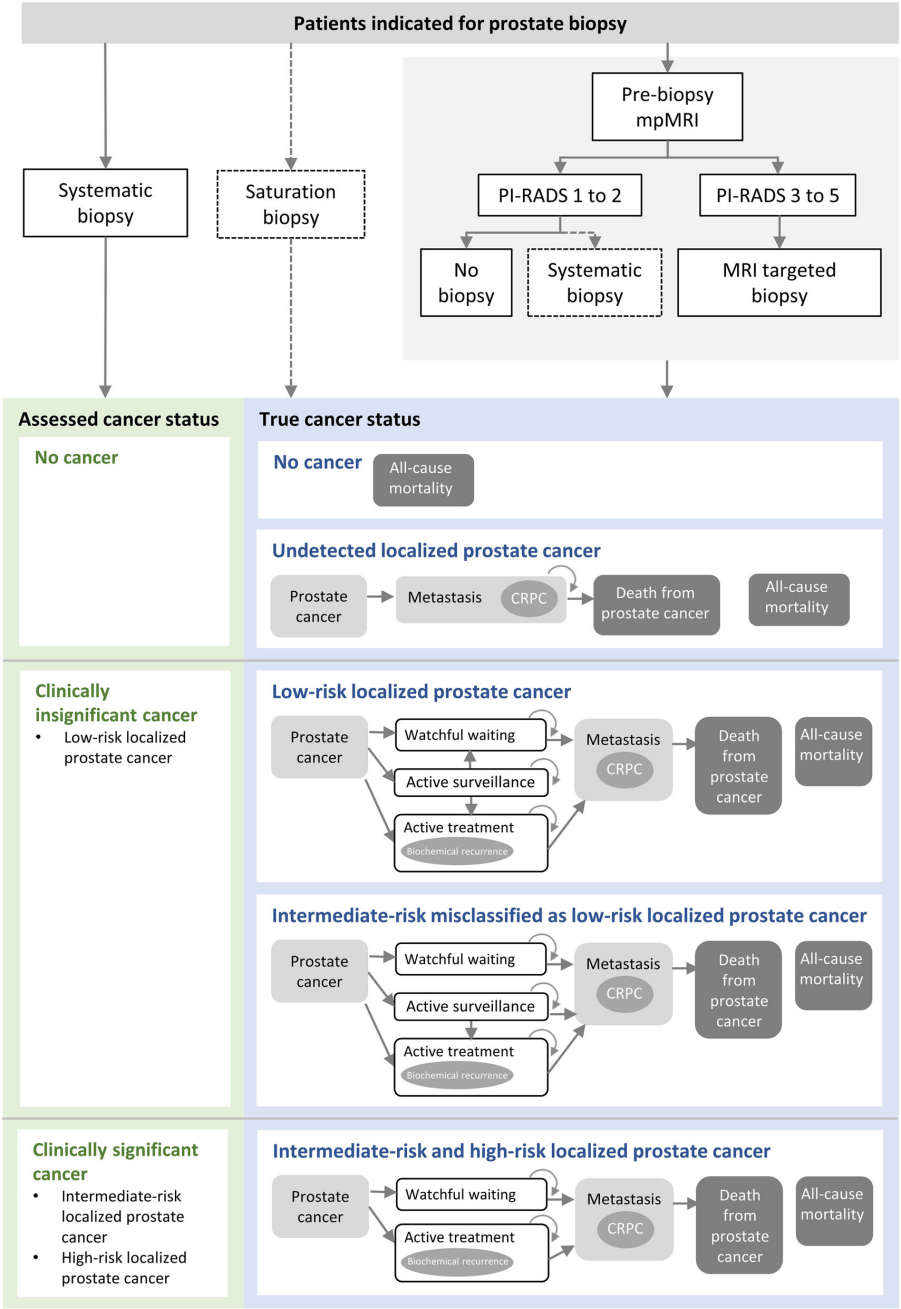
Simplified schema of model structure. Abbreviations: CRPC, castration-resistant prostate cancer; mpMRI, multiparametric magnetic resonance imaging; PI-RADS, Prostate Imaging – Reporting and Data System scores. Notes. 1. MRI targeted biopsy refers to the administration of MRI targeted biopsy combined with systematic biopsy following a positive mpMRI. 2**.** Diagnostic tests used only in repeat biopsy settings (i.e. as second or third biopsy) are represented in dotted lines.

A model combining a decision tree and Markov sub-models was developed in consultation with local clinicians to ensure face validity (see Fig. 1). The decision tree described the detection of prostate cancer and evaluated the likelihood of men classified as having no cancer, or localized prostate cancer of various risk levels conditional on their true disease status. The Markov sub-models described disease progression conditional upon the risk level of the detected localized prostate cancer. These sub-models included the natural history of localized prostate cancer, downstream management strategies and death.

The model was simulated over 20 years using annual cycle length, in line with the estimated life expectancy for men at age 65 years in Singapore [28]. During the simulated time horizon, patients moved through the model based on different transition probabilities to accrue costs and effects for each diagnostic strategy.

The outcome measure of the model was quality-adjusted life years (QALYs). Incremental cost-effectiveness ratios (ICERs) were calculated from pairwise comparisons of incremental costs and QALYs of the six diagnostic strategies. An annual discounting rate of 3% was applied to both costs and effectiveness. All analyses were conducted from the Singapore healthcare system perspective, and all costs were in 2020 United States (US) dollars ($, USD1 = SGD1.36) [29].

The model was built using TreeAge Pro 2018 R2.0 (TreeAge Software, Inc., MA). Analyses were performed using Monte Carlo simulation (seed number set at 16).

### Model input parameters

Model input parameters were obtained from published data where possible and available. To overcome the dearth of real-world local data on downstream management pathways and resources, distribution of care strategies, and distribution of localized prostate cancer across cancer risks, a survey was co-developed and administered to clinician experts from October 2018 to January 2019. Each of the five local public healthcare institutions offering MRI targeted biopsy, and a specialty cancer center, was represented by a clinician expert to provide and coordinate inputs from their practice setting. A total of six responses were received. Clinician experts that provided inputs included urologists, radiologists, and medical oncologist.

#### Natural history and management strategies

Based on the number of patients suspected of prostate cancer referred to public healthcare institutions for the first biopsy in the past 5 years, the estimated prevalence of prostate cancer in men referred for the first biopsy was 37.7%. The risk of disease progression from localized prostate cancer to metastasis and the risk of death depends on age, tumor risk, and prostate cancer management strategy [30]. The model used the European Association of Urology (EAU) risk groups for localized prostate cancer – low-risk, intermediate-risk, and high-risk prostate cancer defined by PSA levels, International Society of Urology Pathology (ISUP) grades, or clinical tumor categories [16]. Low-risk prostate cancers were clinically insignificant, while intermediate or high-risk prostate cancers were clinically significant [31].

Conventional prostate cancer management strategies considered in the model included active surveillance which involved active disease monitoring and potentially curative therapy if the cancer progressed [9]; watchful waiting which is palliative in nature [16]; and active treatment involving radical prostatectomy or radiotherapy with or without androgen deprivation therapy [16]. In the model, after a biopsy-confirmed diagnosis of localized prostate cancer, management strategies were assigned based on the patient’s risk stratification, disease status, and whether the treatment intent was curative or palliative (see Additional file 1 for distribution of care strategies for localized prostate cancer of various risks). Upon detection of intermediate or high-risk localized prostate cancer, patients experienced watchful waiting, active treatment, metastasis, or death; low-risk localized prostate cancer patients including true low-risk or misclassified intermediate-risk experienced watchful waiting, active treatment, active surveillance, metastasis, or death. Patients on active surveillance remained on the same management strategy unless prompted by disease progression to undergo active treatment, or transition to watchful waiting from age of 75 years, whichever earlier [16] .

Undiagnosed patients and patients on active surveillance were assumed to follow progression rates associated with watchful waiting [30]. Patients with true intermediate-risk cancer but misclassified as low-risk cancer received treatments for low-risk localized prostate cancer but followed progression rates for intermediate-risk localized prostate cancer. Only patients with misclassified low-risk prostate cancer on active surveillance were likely to experience metastasis before they switched to active treatment. In line with clinician experts’ inputs, an estimated 50% of patients with misclassified low-risk prostate cancer in the active surveillance cohort switched to active treatment every year. Patients receiving radical prostatectomy and radiotherapy experienced the same progression risk [16]. Patients whose cancer diagnoses were missed by biopsy remained under observation or watchful waiting at age of 75 years and did not receive any active treatment until onset of metastatic disease.

A proportion of patients receiving active treatment could experience biochemical recurrence to trigger further treatments such as salvage treatment [32]. When symptomatic metastases developed, they either received active or palliative care. Some patients with metastatic disease may further progress to castration-resistant prostate cancer (CRPC) despite hormonal therapy with or without the early use of docetaxel or novel oral hormonal agents in the castrate-sensitive state, necessitating the use of other CRPC therapies such as abiraterone, enzalutamide, radium-223, docetaxel and cabazitaxel (see Additional file 2 for distribution of treatments for metastatic cancer and castration-resistant prostate cancer and Additional file 3 for percentage of treatment-related complications).

All prostate cancer-related deaths were assumed to occur following metastatic disease. In any health state, men could die from causes other than prostate cancer by applying the general population’s all-cause mortality rates for resident males [33].

#### Model validation

To ensure face validity, inputs from surveyed clinician experts provided insights to diagnostic and management pathways for prostate cancer, model inputs and assumptions used in the model. For external validity, 15-year overall survival rates from the PREDICT Prostate multivariate model were compared with the current model’s predicted overall survival output (see Additional file 4 for more details on this comparison) [34].

#### Diagnostic performance and uptake

Table 2 shows the diagnostic performance of various tests and the estimated biopsy uptake rates in local clinical practice. Diagnostic performance of the various tests varied in initial and repeat biopsy settings due to differing prostate cancer risks [23, 35]. Patients with positive biopsy findings had their disease staged and received appropriate management. The model assumed that only patients who were truly intermediate-risk could be susceptible to be misclassified and managed as low-risk. In negative biopsies, all false negative cases were assumed to have persistently elevated PSA level, while an estimated 75% of those with true negative results remained clinically suspicious for prostate cancer. These were indications for a subsequent biopsy a year later. The distribution of patients with localized prostate cancer of varying risk was 31% low risk, 44% intermediate risk, and 25% high risk (see Additional file 5 for more details on this distribution).

**Table 2:** Diagnostic performance of diagnostic tests and uptake of subsequent biopsy

Diagnostic test	Uptake	True localized cancer status	Diagnostic test findings	Sources
mpMRI	100%	No cancer	• No suspicion of cancer or suspicion of low-risk cancer: 50% • Suspicion of low-risk cancer: 17% • Suspicion of intermediate or high-risk cancer: 50%	NICE, 2019; Brown et al., 2018
		Low-risk cancer	• No suspicion of cancer or suspicion of low-risk cancer: 44% • Suspicion of low-risk cancer: 16% • Suspicion of intermediate or high-risk cancer: 56%	
		Intermediate or high-risk cancer	• No suspicion of cancer or suspicion of low-risk cancer: 13% • Suspicion of low-risk cancer: 5% • Suspicion of intermediate or high-risk cancer: 87%	
First biopsy: systematic biopsy without prior mpMRI	100%	Low-risk cancer	• P (Low|Low): 35%	Brown et al., 2018; Drost et al., 2019
		Intermediate cancer	• P (Low|Intermediate): 17% • P (Intermediate|Intermediate): 59%	
		High-risk cancer	• P (High|High): 100%	
First biopsy: MRI targeted biopsy after a suspicious mpMRI result	100%	Low-risk cancer	• P (Low|Low): 35%	
		Intermediate cancer	• P (Low|Intermediate): 8% • P (Intermediate|Intermediate): 79%	
		High-risk cancer	• P (High|High): 100%	
Second biopsy: systematic biopsy following a negative systematic biopsy	71%	Low-risk cancer	• P (Low|Low): 45%	
		Intermediate cancer	• P (Low|Intermediate): 10% • P (Intermediate|Intermediate): 35%	
		High-risk cancer	• P (High|High): -	
Second biopsy MRI targeted biopsy following a suspicious mpMRI result and no cancer on prior systematic biopsy	71%	Low-risk cancer	• P (Low|Low): 45%	
		Intermediate cancer	• P (Low|Intermediate): 6% • P (Intermediate|Intermediate): 88%	
		High-risk cancer	• P (High|High): -	
Third biopsy: saturation biopsy	71%	Low-risk cancer	• Sensitivity: 95% • Specificity: 100%	Expert opinion
		Intermediate cancer	• Sensitivity: 95% • Specificity: 100%	
		High-risk cancer	• Sensitivity: 95% • Specificity: 100%	

***Abbreviation***: *mpMRI* multi-parametric magnetic resonance imaging

Notes

1. MRI targeted biopsy refers to the administration of MRI targeted biopsy combined with systematic biopsy following a positive mpMRI.

2. P (Low|Low): probability of detecting low-risk cancer given that low-risk cancer exists; P (Low|Intermediate): probability of detecting low-risk cancer given that intermediate-risk cancer exists; P (Intermediate|Intermediate): probability of detecting intermediate-risk cancer given that intermediate-risk cancer exists; P(High|High): probability of detecting high-risk cancer given that high-risk cancer exists.

3. Estimated uptake rates were from surveyed clinician experts from public healthcare institutions.

4. From the surveyed clinician experts, based on the number of patients suspected of prostate cancer referred to public healthcare institutions for the first biopsy in the past five years, the estimated prevalence of prostate cancer in men referred for the first biopsy was 37.7%.

5. The uncertainty of model inputs was explored by simultaneously and randomly sampling the parameters from assigned distributions – beta distribution for health utilities values and multivariate normal distributions using Cholesky decomposition matrix for parameters characterizing disease progression.

#### Health utilities

A QALY is an outcome measure derived by adjusting the length of time by quality of life measured in health utilities on a scale of 0 (death) to 1 (perfect health) [36]. Table 3 shows health utilities inputs in the model. Utilities for men with the modelled starting age of 65 years with no diagnosed cancer was taken from a multi-country population norms study using the EQ-5D-3L [37]. The model considered utility decrements associated with age [38], utilities associated with localized prostate cancer of various risk levels and metastases [39], and impact of biopsies on utilities [31, 35]. Varying complication rates arising from saturation or systematic biopsies led to different utility decrements [31, 35]. Patients with undetected cancer or on active surveillance were assumed to have the same utilities as patients on watchful waiting. Utilities of patients with CRPC were lower than those without CRPC assuming no substantial decline in utilities until CRPC developed [40]. Metastatic patients receiving palliative care were assumed to have the same utilities as those not receiving CRPC care. Health utility benefits associated with active treatment was assumed to be equal for low, intermediate, and high-risk localized cancers, and remained constant until metastasis occurred. Treatment complications and biochemical recurrence were associated with utility decrements [41, 42].

**Table 3:** Health utilities weights and decrements in the model

Parameter	Value (range)	Source	Other remarks
**Cancer health state**			
No cancer	0.85 (0.83 to 0.86)	Clemens et al. 2014	–
Low-risk localized prostate cancer	0.84 (0.836 to 0.844)	Stewart et al. 2005	At baseline, the utility value for patients at the metastasis state is 0.67. The utility is reduced to 0.4 when they progress to castration-resistant prostate cancer. In the last two months before death, metastatic patients’ utility was assumed to increase to 0.67 as they would receive palliative care to maintain quality of life.
Intermediate-risk localized prostate cancer	0.81 (0.803 to 0.817)		
High-risk localized prostate cancer	0.71 (0.701 to 0.719)		
Metastasis	0.67 (0.660 to 0.680)		
Utility benefit of active treatment	0.01	Korfage et al. 2005	Utility benefit was derived from the difference before and after the active treatment in Korfage et al. (2005). Utility values at the active treatment health state: 0.85 for low-risk prostate cancer; 0.82 for intermediate-risk; 0.72 for high-risk.
Castration-resistant prostate cancer	0.40 (0.3 to 0.5)	Bayoumi et al. 2000	–
**Utilities decrement**			
Age	0.0002587 + 0.0000332* (age^2-(age-1)^2)	Ara and Brazier 2010	–
Biopsy complication – saturation biopsy	0.00677 (0.00577 to 0.00769)	Brown et al. 2018	Utility change post-saturation biopsy of −0.176 (−0.15 to −0.2) was obtained from PROMIS individual patient data: 0.176*2/52 = 0.00677; Utility change post-systematic biopsy: 0.101*2/52 = 0.00388.
Biopsy complication – systematic biopsy	0.00388 (0.00349 to 0.00427)	NICE (2019)	
Treatment complication – erectile dysfunction	0.1	Krahn et al. 2003	Erectile dysfunction and urinary urgency are lifelong complications.
Treatment complication – urinary urgency	0.06		
Treatment complication – bowel problem	0.11		
Recurrence	0.0206 (0 to 0.08893)	Ramsay et al. 2012	Utility value for chemical recurrence reported in Ramsay et al. (2012) is 0.73 for patients with high-risk prostate cancer, based on a baseline utility for men without cancer of 0.95, which was higher than that used in our model (0.85). Utility decrement associated with chemical recurrence applied in the model was therefore calculated by deducting the baseline utility of high-risk prostate cancer by the adjusted value for chemical recurrence: 0.71–0.73*(0.85/0.9) = 0.0206.

Notes

1. The uncertainty of model inputs was explored by simultaneously and randomly sampling the parameters from assigned distributions – beta distribution for health utilities values and multivariate normal distributions using Cholesky decomposition matrix for parameters characterizing disease progression.

#### Costs

In line with a healthcare system perspective, only direct medical resources were considered. Table 4 shows that cost inputs included charges associated with the diagnostic strategies, management and treatment of prostate cancer, and management of complications.

**Table 4:** Cost inputs (in USD) in the model

Item	Mean value	Unit	Source	Other remarks
**Cost inputs for diagnosis**				
Urology visit	82	Per visit	Public healthcare institutions	–
PSA test	41	Per test	Public healthcare institutions	–
Systematic biopsy	1108	Per session	MOH Casemix and Subvention System (2017)	–
Saturation biopsy	1563	Per session	MOH Casemix and Subvention System (2017)	–
mpMRI	827	Per session	Public healthcare institutions	–
MRI targeted biopsy (inclusive of mpMRI, MRI-US fusion targeted biopsy and systematic biopsy)	2223	Per session	Public healthcare institutions	–
**Cost inputs for management and treatment strategy for localized prostate cancer**				
Observation	122	Per year	Public healthcare institutions	1 visit to the urologist and PSA testing per year
Watchful waiting	163	Per year	Public healthcare institutions	1.5 visits to the urologist and PSA testing per year
Radical prostatectomy	17,023	Per episode	MOH Casemix and Subvention System (2017)	Laparoscopic surgery
Radiotherapy (curative)	18,382	40 treatment sessions	Public healthcare institutions	External beam only, with image-guided radiotherapy
Androgen deprivation therapy	439	3-monthly	Drug Utilisation Data (2017)	In curative treatment, androgen deprivation therapy included only Luteinizing hormone-releasing hormone (LHRH) agonist. The 3-monthly cost of LHRHa was the weighted mean selling price calculated based on all LHRHa (leuprorelin acetate, goserelin, leuprorelin and triptorelin) in Drug Utilization Data (2017).
**Cost inputs for management and treatment strategy for metastasis and castration-resistant prostate cancer**				
Metastatic care - first line therapy	2194	Per year	• Survey of local experts • Drug Utilization Data (2017) • MOH Casemix and Subvention System (2017)	–
Metastatic care – second line therapy	18,349	Per year		
Treatment for castration-resistant prostate cancer	26,458	Per year		
Hospice care	202	Per day	Assisi Hospice	–

***Abbreviations*:***MRI* magnetic resonance imaging, *PSA* prostate-specific antigen, *US* ultrasound, *USD* US dollar

**Note:**

1. After positive mpMRI, MRI targeted biopsy (using MRI-US fusion targeted biopsy) is administered with systematic biopsy. The total cost of MRI targeted biopsy when administered with systematic biopsy (inclusive of mpMRI) is $2223 per session.

2. The uncertainty of model inputs was explored by simultaneously and randomly sampling the parameters from assigned distributions – beta distribution for health utilities values and multivariate normal distributions using Cholesky decomposition matrix for parameters characterizing disease progression.

### Sensitivity analyses

One-way sensitivity analyses were conducted over the range of predefined values for specific model parameters using the reported 95% confidence intervals from published literature or ± 10% of point estimate. Except for costs of mpMRI and biopsies in the evaluated diagnostic strategies, costs were considered known parameters and were not evaluated in the one-way sensitivity analyses. If more than two strategies were considered cost-effective, one-way sensitivity analyses were performed for the pair of strategies with the highest ICER in the deterministic base case analyses. The least costly strategy was used as the reference strategy for the pairwise comparison.

Multivariate probabilistic sensitivity analyses with 1000 second-order Monte Carlo simulations (50,000 first-order simulation trials) were performed. The uncertainty of model inputs was explored by simultaneously and randomly sampling the parameters from assigned distributions. Cost-effectiveness acceptability curves presented the cost-effectiveness probability of each diagnostic strategy over a range of willingness-to-pay (WTP) thresholds.

## Results

### Base case analyses

**Fig. 2:**
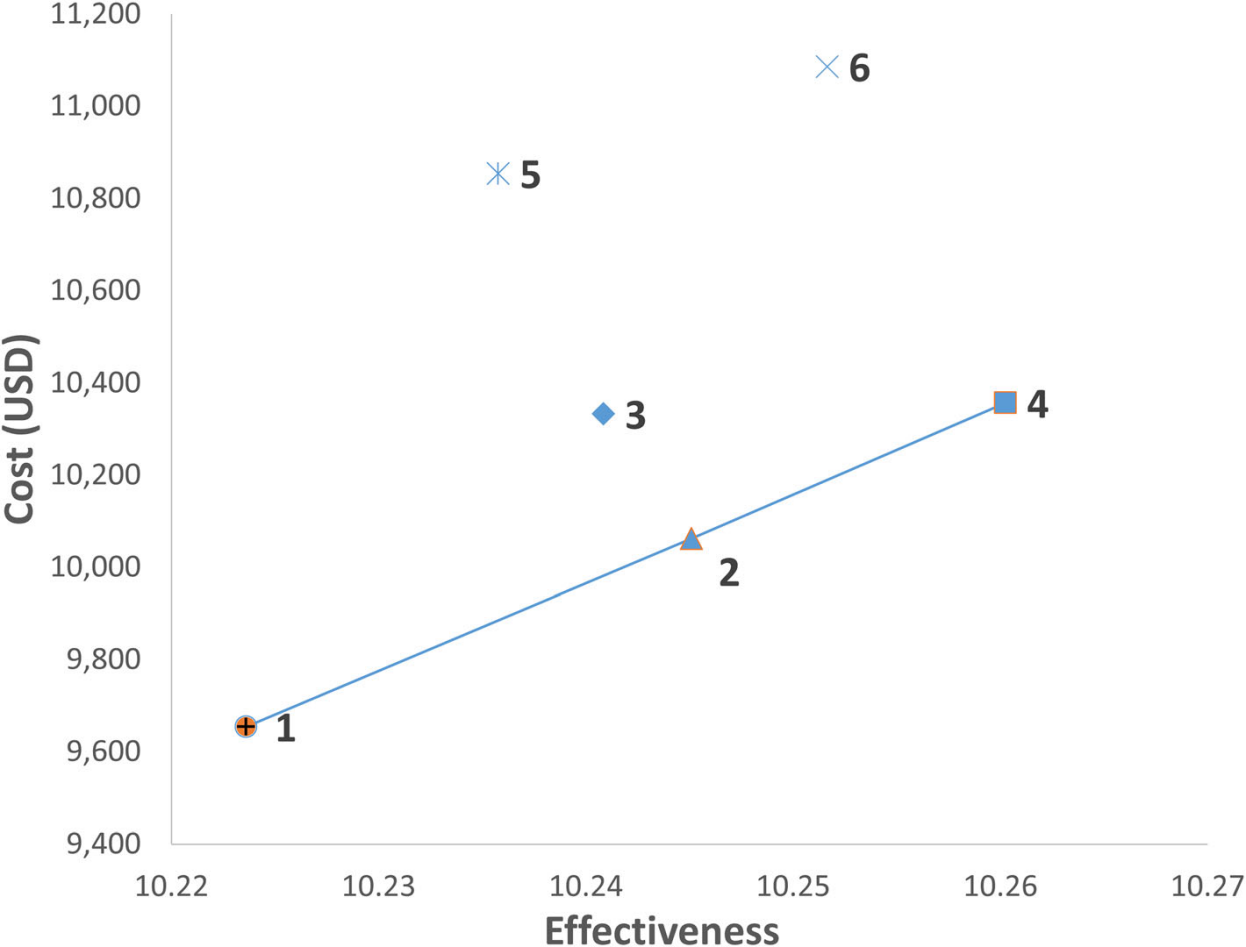
Cost-effectiveness plane of base case analyses. Abbreviations: mpMRI, multiparametric magnetic resonance imaging; MRI, magnetic resonance imaging; QALY, quality-adjusted life years; USD, US dollars. Notes. 1. Strategy 1: MRI targeted biopsy. Strategy 2: MRI targeted biopsy ➔ Systematic biopsy. Strategy 3: MRI targeted biopsy ➔ Saturation biopsy. Strategy 4: MRI targeted biopsy ➔ Systematic biopsy ➔ Saturation biopsy. Strategy 5: Systematic biopsy ➔ MRI targeted biopsy. Strategy 6: Systematic biopsy ➔ MRI targeted biopsy ➔ Saturation biopsy. 2. MRI targeted biopsy refers to the administration of MRI targeted biopsy combined with systematic biopsy following a positive mpMRI. 3. Strategies 1, 2, 4 are on the cost-effectiveness frontier, indicating that they achieve the most QALY per USD spent; Strategies 3, 5, 6 are dominated as they are more costly and gave fewer QALYs.

Figure 2 showed strategies 1, 2 and 4 on the cost-effectiveness plane, indicating that these strategies achieved the most QALYs per USD spent. Strategy 1 had the lowest cost, while Strategies 2 and 4 gave ICERs ranging from US$18,975 to US$19,458 when compared to Strategies 1 and 2 respectively (see Table 5). When Strategy 4 was compared to Strategy 1, the ICER was US$19,175. Strategies 1, 2, 4 involved MRI targeted biopsy used in the biopsy-naïve patients in the initial biopsy setting and were considered cost-effective, assuming a WTP threshold of US$20,000. On the other hand, strategies that involved MRI targeted biopsy in the repeat biopsy setting (Strategies 5 and 6) were dominated, incurring more costs with less QALY gain. Similarly, Strategy 3 was dominated, suggesting saturation biopsy, when used, should be reserved as the last option within a testing sequence.

**Table 5:** Base case analyses of diagnostic strategies

Diagnostic strategy	Comparator	20 years horizon					25 years horizon	35 years horizon
		Costs (USD)	Incremental costs	Effectiveness (QALYs)	Incremental effectiveness	ICER (cost in USD per QALY gained)	ICER (cost in USD per QALY gained)	
Strategy 1*: MRI targeted biopsy	–	9655	–	10.2236	–	–	–	–
Strategy 2*: MRI targeted biopsy ➔ Systematic biopsy	Strategy 1	10,062	408	10.2451	0.0215	18,975	16,211	14,084
Strategy 3: MRI targeted biopsy ➔ Saturation biopsy	Strategy 2	10,333	271	10.2408	−0.0042	Dominated by Strategy 2		
Strategy 4*: MRI targeted biopsy ➔ Systematic biopsy ➔ Saturation biopsy	Strategy 2	10,357	295	10.2602	0.0152	19,458	15,915	14,106
Strategy 5: Systematic biopsy ➔ MRI targeted biopsy	Strategy 4	10,855	498	10.2358	−0.0245	Dominated by Strategy 4		
Strategy 6: Systematic biopsy ➔ MRI targeted biopsy ➔ Saturation biopsy	Strategy 4	11,086	729	10.2516	−0.0086	Dominated by Strategy 4		

***Abbreviations***: *ICER* incremental cost-effectiveness ratio, *mpMRI* multi-parametric magnetic resonance imaging, *QALY* quality-adjusted life year; USD, US dollar

**Note**:

1. MRI targeted biopsy refers to the administration of MRI targeted biopsy combined with systematic biopsy following a positive mpMRI.

2. Arrow (➔) refers to next sequence of diagnostic test following a negative biopsy result

3. The diagnostic strategies are organized from the least costly to most mostly strategy based on the analyses for 20-year time horizon. The incremental cost and effectiveness of each strategy is calculated by comparing against the preceding strategy that is not dominated. A dominated strategy is more costly and less effective than the strategy in the immediately preceding row. The strategies with asterisk (*) are not dominated; those without asterisk are dominated.

4. When Strategy 4 was compared against Strategy 1, the ICER was US$19175.

### Sensitivity analyses

**Fig. 3:**
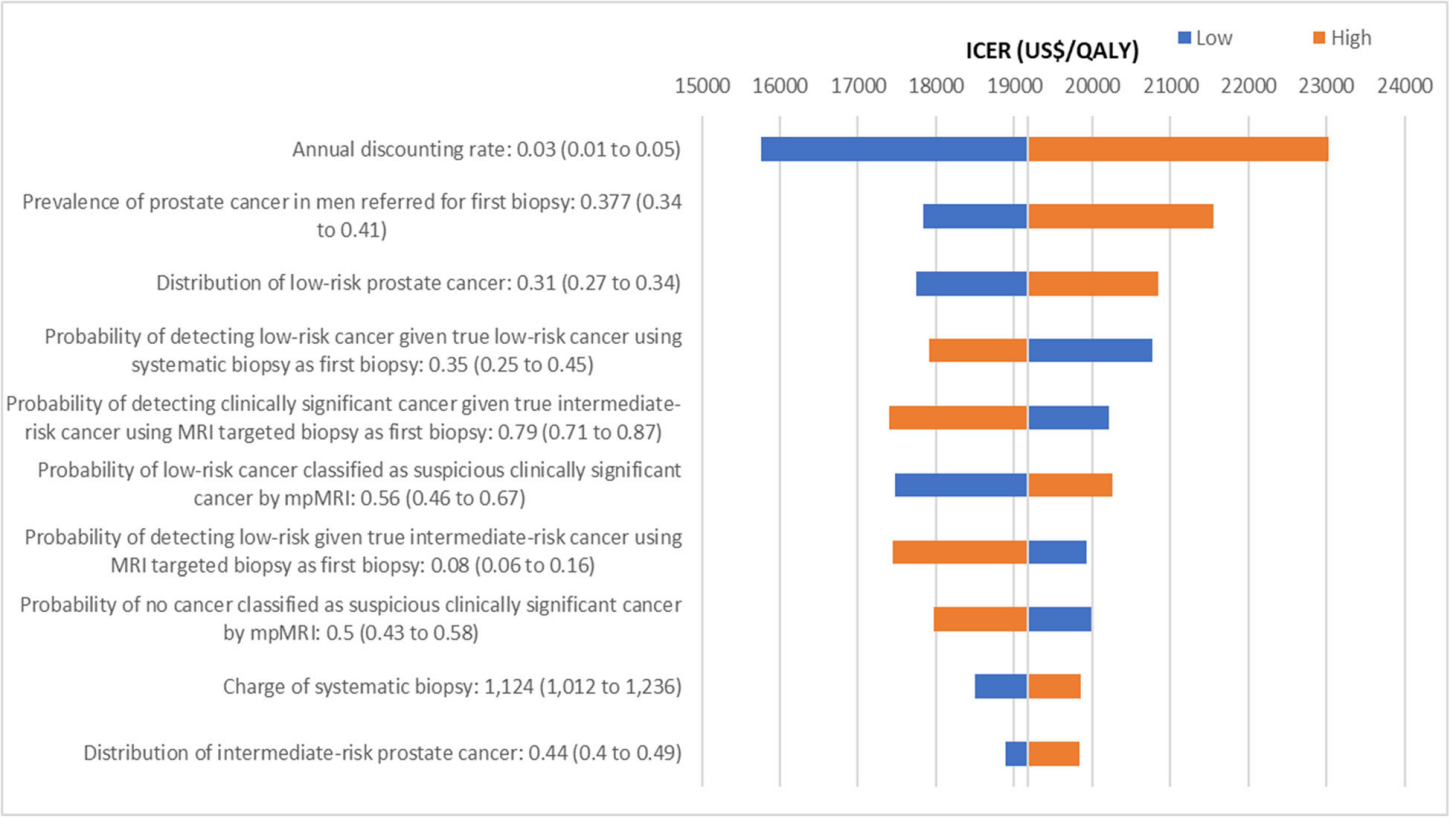
ICER tornado diagram for Strategy 4 vs Strategy 1 (top 10 drivers). Abbreviations: ICER, incremental cost-effectiveness ratio; MRI, magnetic resonance imaging; mpMRI, multi-parametric magnetic resonance imaging; PSA, prostate-specific antigen. Notes. 1. Strategy 1: MRI targeted biopsy. Strategy 4: MRI targeted biopsy ➔ Systematic biopsy ➔ Saturation biopsy. 2. MRI targeted biopsy refers to the administration of MRI targeted biopsy combined with systematic biopsy following a positive mpMRI. 3. Blue bars denote the ICERs when the parameter’s lower bound limit is tested; red bars denote the ICERs when the parameter’s upper bound limit is tested.

One-way sensitivity analyses were performed for Strategy 4 compared with Strategy 1 as the reference strategy as Strategy 1 had the lowest cost. Figure 3 showed that ICER was most sensitive to annual discounting rate, prevalence of prostate cancer in men referred for first biopsy, probability of detecting low-risk cancer given true low-risk cancer using systematic biopsy as first biopsy, and the probability of low-risk cancer classified as suspicious clinically significant cancer by mpMRI (see Additional file 6 for summary of one-way sensitivity analyses parameters and results of Strategy 4 vs Strategy 1). All ICERs remained between US$15,755 to US$23,022 per QALY gained when model parameters were varied (see Additional file 7 for full chart of ICER tornado diagram for Strategy 4 vs Strategy 1).

**Fig. 4:**
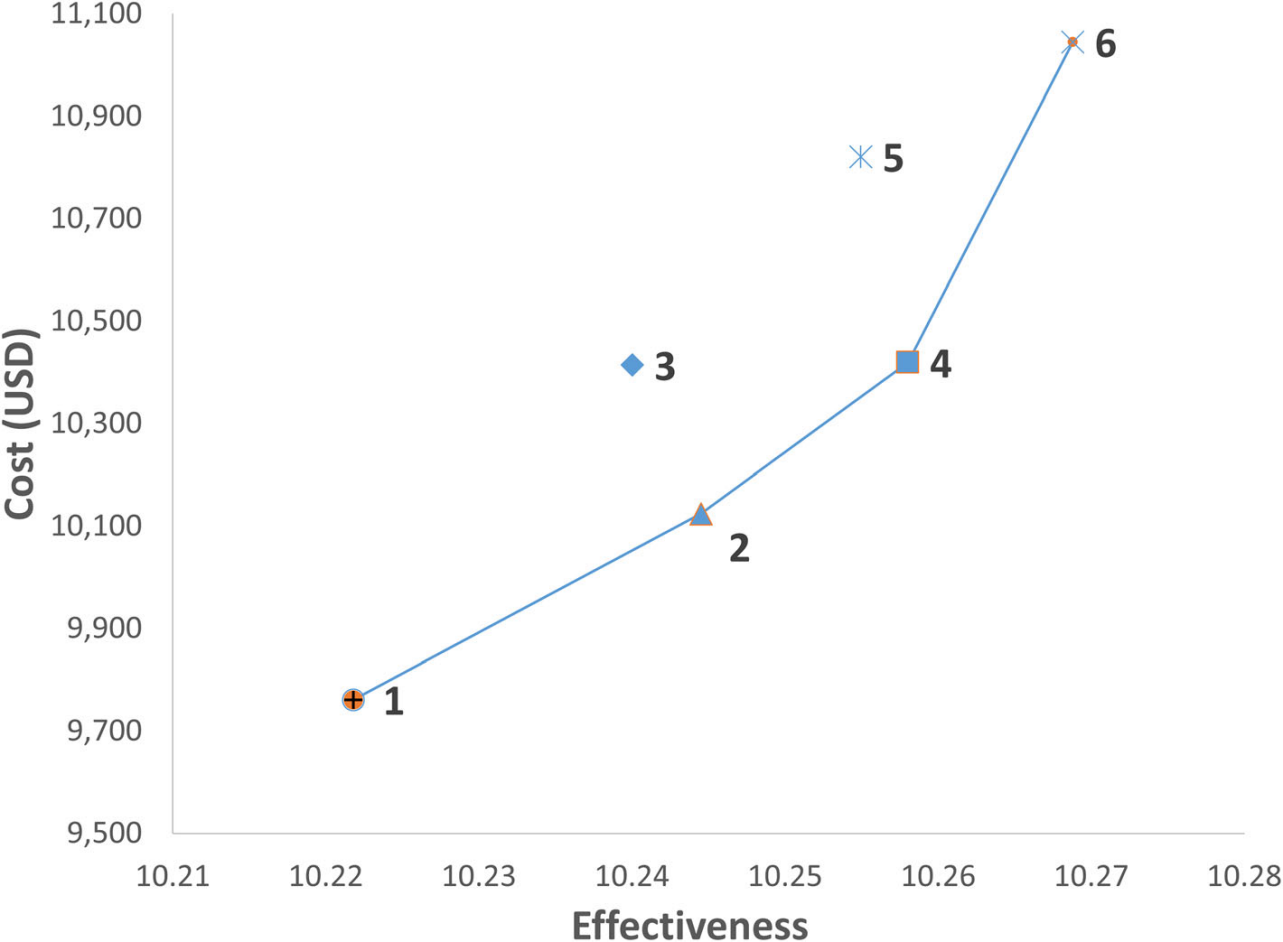
Cost-effectiveness plane of probabilistic sensitivity analyses. Abbreviations: mpMRI, multiparametric magnetic resonance imaging; MRI, magnetic resonance imaging; QALY, quality-adjusted life years; USD, US dollars. Notes. 1. Strategy 1: MRI targeted biopsy. Strategy 2: MRI targeted biopsy ➔ Systematic biopsy. Strategy 3: MRI targeted biopsy ➔ Saturation biopsy. Strategy 4: MRI targeted biopsy ➔ Systematic biopsy ➔ Saturation biopsy. Strategy 5: Systematic biopsy ➔ MRI targeted biopsy. Strategy 6: Systematic biopsy ➔ MRI targeted biopsy ➔ Saturation biopsy. 2. MRI targeted biopsy refers to the administration of MRI targeted biopsy combined with systematic biopsy following a positive mpMRI. 3. Strategies 1, 2, 4, 6 are on the cost-effectiveness frontier, indicating that they achieve the most QALY per USD spent; Strategies 3, 5 are dominated as they are more costly and gave fewer QALYs.

**Fig. 5:**
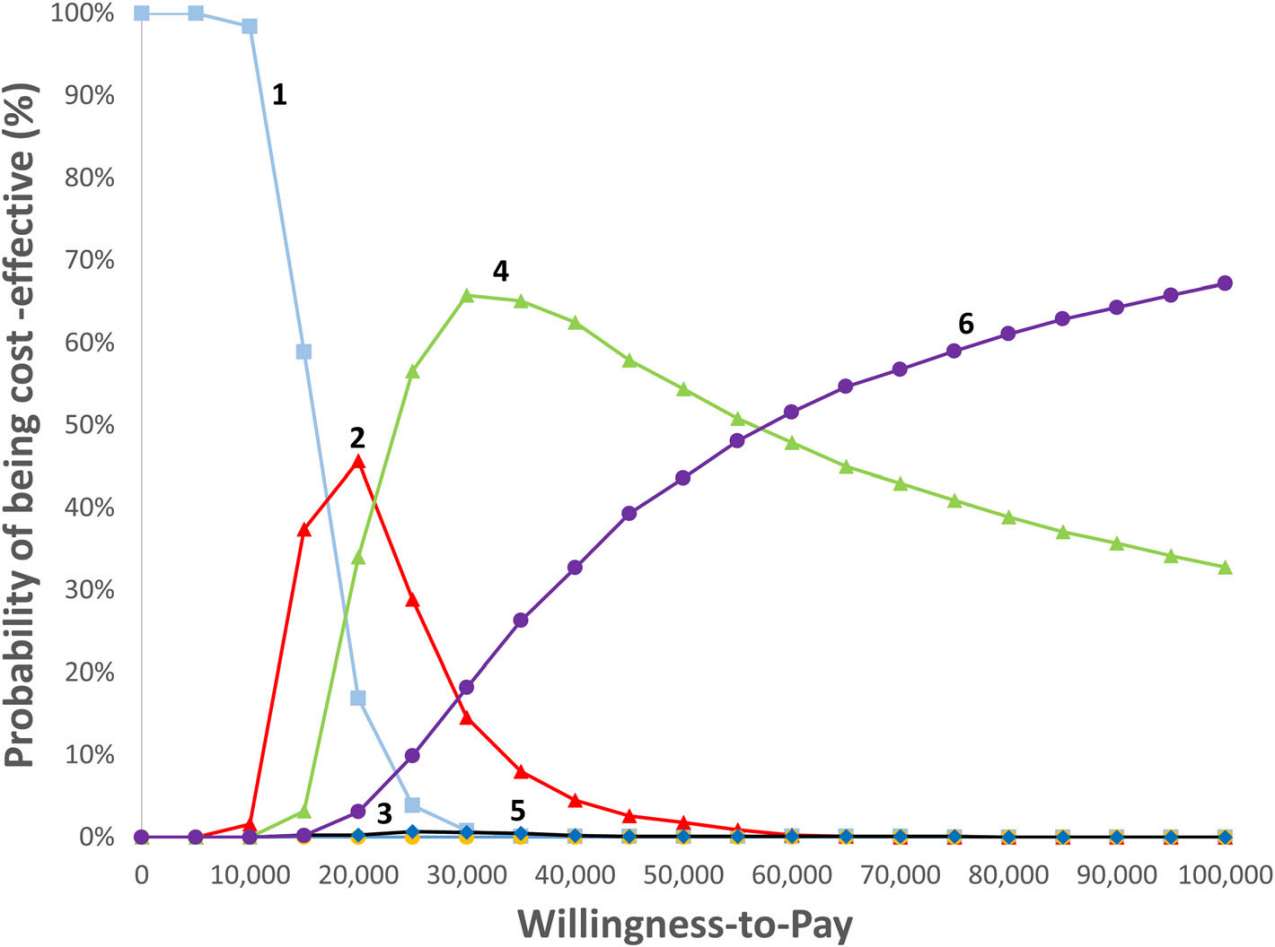
Cost-effectiveness acceptability curves. Abbreviations: mpMRI, multiparametric magnetic resonance imaging; MRI, magnetic resonance imaging; QALY, quality-adjusted life years; USD, US dollars. Notes. 1. Strategy 1 (shown in blue squares): MRI targeted biopsy. Strategy 2 (shown in red triangles): MRI targeted biopsy ➔ Systematic biopsy. Strategy 3 (shown in yellow circles): MRI targeted biopsy ➔ Saturation biopsy. Strategy 4 (shown in green triangles): MRI targeted biopsy ➔ Systematic biopsy ➔ Saturation biopsy. Strategy 5 (shown in blue diamonds): Systematic biopsy ➔ MRI targeted biopsy. Strategy 6 (shown in purple ovals): Systematic biopsy ➔ MRI targeted biopsy ➔ Saturation biopsy. 2. MRI targeted biopsy refers to the administration of MRI targeted biopsy combined with systematic biopsy following a positive mpMRI

Probabilistic sensitivity analyses in Fig. 4 found that Strategies 1, 2, 4, and 6 were on the cost-effectiveness frontier, while Strategies 3 and 5 remained dominated. The ICERs for Strategies 1, 2, 4, and 6 ranged from US$15,990 to US$58,097 (see Additional file 8 for probabilistic sensitivity analyses of all strategies for base case). The cost-effectiveness acceptability curves in Fig. 5 confirmed that Strategy 1 was the least costly, and Strategy 2 was potentially cost-effective at WTP of US$20,000 and Strategy 4 at WTP of US$30,000. However, the probability of Strategy 2 being cost-effective was less than 50%. Strategy 6 could only be considered cost-effective when WTP increased to US$60,000.

## Discussion

This study found that all strategies including MRI targeted biopsy in the initial biopsy setting were cost-effective except when saturation biopsy was used immediately as a second biopsy. Many published cost-effectiveness analyses only evaluated MRI targeted biopsy as a first biopsy compared with systematic transrectal ultrasound-guided (TRUS) biopsy based on a single biopsy protocol [43–46]. With MRI targeted biopsy becoming more commonly used [16, 17], a more pertinent resource allocation question should be – what are the cost-effective ways to deploy MRI targeted biopsy, given the many possible diagnostic sequences within a clinical care pathway. By varying the position of MRI targeted biopsy in six diagnostic strategies, single and multiple biopsy strategies containing MRI targeted biopsy could address how its introduction in a testing strategy could impact its cost-effectiveness. To the best of our knowledge, no other study has evaluated the cost-effectiveness of MRI targeted biopsy within a testing sequence.

This study also incorporated relevant clinical considerations to estimate long-term costs and effectiveness of various diagnostic strategies in single and multiple biopsy settings. Diagnostic accuracy inputs on MRI targeted biopsy for different risk groups were drawn mainly from a recent Cochrane review which comprehensively reviewed the evidence on MRI targeted biopsies based on 17 studies in initial biopsy setting and 8 studies in the repeat biopsy setting [23]. This avoids mixing cost and health impact of detecting clinically significant and clinically insignificant prostate cancer [45, 47]. Downstream management strategies were comprehensively modelled to ensure that diagnostic outcomes and follow-up care included expensive CRPC drug therapies such as abiraterone, enzalutamide, and cabazitaxel. While early detection of treatable clinically significant cancer is the aim of any prostate biopsy, new developments in downstream management of patients with detected cancer or those with persistent clinical suspicion of prostate cancer despite previous negative biopsies can potentially create an undesirable situation where their consequent management accrue higher costs and lower utilities.

The consistently favorable results of Strategies 1, 2 and 4 showed that early use of MRI targeted biopsy as a first biopsy was potentially cost-effective but could be influenced by when saturation biopsy was used. Strategy 3 where saturation biopsy was the second biopsy was shown to be dominated, whereas Strategy 4 had an ICER of US$19,458 when saturation biopsy was used as the third biopsy, implying later use of saturation biopsy could improve the cost-effectiveness of MRI targeted biopsy in the initial biopsy setting. Despite high sensitivity of 95% in detecting localized prostate cancer, saturation biopsy gave greater utility decrements and costs arising from higher complication rates than systematic biopsy.

The discordant results for Strategy 6 in the deterministic base case analyses and probabilistic sensitivity analyses could be explained by the modest difference in the incremental effectiveness between Strategy 4 and Strategy 6, and the multiplicative effect of input parameters in a nonlinear model. In both initial and repeat biopsies, MRI targeted biopsy improved detection of clinically significant cancer and clinically insignificant cancer [23]. Compared to Strategy 4, Strategy 6 had marginally higher detection rate of clinically significant cancer by 0.02%, and clinically insignificant cancer by 0.2% [23]. For strategies with similar diagnostic performance like Strategies 4 and 6, any change in costs and QALYs resulting from a complex nonlinear model with multiplicative effects of input parameters can introduce further uncertainty in the cost-effectiveness result. As probabilistic sensitivity analyses are preferred for estimating mean costs and outcomes in nonlinear models [48, 49], the results lend support for using MRI targeted biopsy in the initial and repeat biopsy settings. Although Strategy 1, a single biopsy protocol, is least costly, it has limited applicability in cases with persistently high clinical suspicion.

Despite a lack of published literature comparing diagnostic strategies that varied position of the MRI targeted biopsy, published literature found greater inconsistency in the cost-effectiveness of MRI targeted biopsy in the repeat biopsy setting. MRI targeted biopsy compared with TRUS systematic biopsy in the initial biopsy setting were consistently cost-effective [43–47, 50]; published literature on MRI targeted biopsy as a second biopsy reported wide ranging results from £5778 per QALY to being dominated when compared with TRUS systematic biopsy [31, 35, 50], indicating less consistent findings in this setting. This could be due to the mounting diagnostic challenge as the yield of clinically significant cancer progressively declines with each subsequent biopsy [51] and the variable downstream care strategies in different practice settings. Recent guidelines also indicated varied strength when recommending MRI targeted biopsy use in repeat biopsy settings [16, 17].

Successful implementation of any diagnostic strategies in a practice setting requires several considerations. The effectiveness of MRI targeted biopsy depends on the quality assurance of mpMRI. Although PI-RADS scores help standardize the acquisition, interpretation and reporting of prostate mpMRI, the learning curve of interpreting mpMRI remains steep and inter-observer differences persist. Radiology practices performing prostate mpMRI should engage in in-house training and continual quality improvement programs to ensure a minimum competency standard is maintained [46, 52]. With evidence supporting prostate mpMRI as a triage in the initial biopsy setting [35], the utilization of mpMRI and its corresponding waiting time are likely to increase but potentially with corresponding reduction in unnecessary biopsies.

This study has several limitations. First, the diagnostic input of MRI targeted biopsy was based upon a review that included all MRI targeted biopsy techniques including cognitive MRI targeted biopsy and in-bore MRI targeting [23]. Variations in these targeted techniques, scanning protocols, varying thresholds for mpMRI positivity to trigger biopsy could contribute to heterogeneity. Despite these sources of heterogeneity, current evidence has not demonstrated clear superiority of one MRI-based biopsy technique over another [16]. Sensitivity analyses performed in this study using published 95% confidence intervals also helped ensure the robustness of the results.

Second, probabilities of metastatic progression and treatment effects were sourced from the SPCG-4 trial which provided up to 18 years’ follow-up data [30, 53]. Although the trial provided good long-term follow-up data, it recruited patients between 1989 and 1999 when PSA testing was not yet widely adopted. Recent improvements in contemporary treatment modalities such as robotic surgery and more precise radiotherapy are expected to give better QALY benefits. The more recent PIVOT trial enrolled men from 1994 to 2002 during the early era of PSA testing but did not report time to progression for each risk group as required by this model [54, 55]. As such, the progression rates of patients receiving radical prostatectomy in the SPCG-4 trial represented a conservative estimate of the treatment modality, while those receiving watchful waiting best approximated the natural history of prostate cancer without treatment [31].

Third, this study did not include a potential diagnostic strategy with two consecutive MRI targeted biopsies due to its low frequency of use in local clinical practice and lack of clarity in the guidelines. This diagnostic strategy could take place if there were concerns that the first MRI targeted biopsy was not optimal due to technical reasons or if the second mpMRI showed changes in the lesion(s) after a negative initial biopsy. Future studies could consider including this strategy when there is more published data or greater clarity in guidelines.

Fourth, model inputs such as the prevalence of prostate cancer in men referred for biopsy, the distribution of localized prostate cancer risks, and allocation of care and treatment strategies were informed through surveys of clinician experts working in the local public healthcare institutions. This was due to a lack of published real-world local data. To ensure representativeness of the findings, each public healthcare institution offering prostate biopsy had at least one urologist providing input. Additional inputs from radiologists were also sought. As model inputs and assumptions were developed for a starting age of 65 years, its findings cannot readily apply to other starting ages without a separate undertaking to ensure rigor and relevance of model input and assumptions.

Fifth, health utility scores were from published literature derived from Australia, Canada, Netherlands, United Kingdom, and USA due to a lack of published local information [39–41, 56, 57]. Given a lack of local published health utilities, these sources present the best available published evidence that met the data needs of the model. While the emotional toll of a cancer diagnosis could adversely impact quality of life, similar utilities between no cancer and localized low-risk prostate cancer were applied based on available published utilities. The small difference is expected given the typically slow-progressing nature of prostate cancer. When varied in one-way sensitivity analyses, they did not materially impact the ICERs (see Additional file 8 for probabilistic sensitivity analyses of all strategies for base case).

## Conclusions

To conclude, this study found Strategy 1, Strategy 2, and Strategy 4 – where MRI targeted biopsy was used in biopsy-naïve patients in the initial biopsy setting – to be cost-effective diagnostic options for prostate cancer. The findings are useful to inform decision making in funding different diagnostic options within the Singapore public healthcare system.

## Supplementary Information

**Additional file 1: Table S1.** Distribution of care strategies for localized prostate cancer of various risks.

**Additional file 2: Table S2.** Distribution of treatments for metastatic cancer and castration-resistant prostate cancer.

**Additional file 3: Table S3.** Percentage of the treatment-related complications.

**Additional file 4: Table S4.** Comparison of overall survival of men from published prostate cancer data and current study’s modelled population.

**Additional file 5: Table S5.** Distribution of patients with localised prostate cancer across risk status.

**Additional file 6: Table S6.** Summary of one-way sensitivity analyses parameters and results - Strategy 4 vs Strategy 1.

**Additional file 7: Fig. S1.** ICER tornado diagram for Strategy 4 vs Strategy 1 (full chart).

**Additional file 8: Table S7.** Probabilistic sensitivity analyses of all strategies for base case.

## Data Availability

The dataset(s) supporting the conclusions of this article are included within the article and (and its additional file(s)).

## Abbreviations

CRPC: Castration-resistant prostate cancer
DRE: Digital rectal examination
EAU: European Association of Urology
ICER: Incremental cost-effectiveness ratio
ISUP: International Society of Urology Pathology
mpMRI: Multiparametric magnetic resonance imaging
MRI: Magnetic resonance imaging
MRI-US: Magnetic resonance imaging - ultrasound
PI-RADS: Prostate Imaging – Reporting and Data System
PSA: Prostate-specific antigen
QALY: Quality-adjusted life year
TRUS: Transrectal ultrasound-guided
US: United States
USD: United States dollar
WTP: Willingness-to-pay
