# Supplementary material for: Cost-effectiveness of MRI targeted biopsy strategies for diagnosing prostate cancer in Singapore
Source: BMC Health Serv Res. 2021 Sep 3;21:909. doi: 10.1186/s12913-021-06916-0 (PMC8414680; doi:10.1186/s12913-021-06916-0)
Supplement: Supplementary file 1 — Additional file 1: Table S1. Distribution of care strategies for localized prostate cancer of various risks. [file 12913_2021_6916_MOESM1_ESM.docx]

**Table S-1. Distribution of care strategies for localized prostate cancer of various risks**

| Detected by | | Care strategy | Distribution | Source |
| --- | --- | --- | --- | --- |
| **Managing low-risk localized prostate cancer** | | | | |
| MRI targeted biopsy | | Watchful waiting | 13% | Survey of local experts |
|  |  | Active surveillance | 63% |  |
|  |  | Active treatment | 25% |  |
| Systematic biopsy | | Watchful waiting | 19% |  |
|  |  | Active surveillance | 53% |  |
|  |  | Active treatment | 28% |  |
| **Managing intermediate-risk localized prostate cancer** | | | | |
| MRI targeted biopsy | | Watchful waiting | 17% | Survey of local experts |
|  |  | Active treatment | 83% |  |
| Systematic biopsy | | Watchful waiting | 13% |  |
|  |  | Active treatment | 87% |  |
| **Managing high-risk localized prostate cancer** | | | | |
| MRI targeted biopsy | Watchful waiting | | 3% | Survey of local experts |
|  | Active treatment | | 97% |  |
| Systematic biopsy | Watchful waiting | | 6% |  |
|  | Active treatment | | 94% |  |

**Note:**

1. MRI targeted biopsy refers to the administration of MRI targeted biopsy combined with systematic biopsy following a positive mpMRI.
