# Supplementary material for: Cost-effectiveness of MRI targeted biopsy strategies for diagnosing prostate cancer in Singapore
Source: BMC Health Serv Res. 2021 Sep 3;21:909. doi: 10.1186/s12913-021-06916-0 (PMC8414680; doi:10.1186/s12913-021-06916-0)
Supplement: Supplementary file 2 — Additional file 2: Table S2. Distribution of treatments for metastatic cancer and castration-resistant prostate cancer. [file 12913_2021_6916_MOESM2_ESM.docx]

Table S-2. Distribution of treatments for metastatic cancer and castration-resistant prostate cancer

| Managing metastasis | Use of care | Dosing regimen | Source |
| --- | --- | --- | --- |
| **First-line therapy for metastatic cancer** | | | |
| Bilateral orchiectomy | 8% | - | Survey of local experts |
| LHRHa | 86% | An example of LHRHa is leuprorelin 11.25mg every 3 months |  |
| Degarelix | 6% | Degarelix 240mg as starting dose, then 80mg as maintenance dose every month |  |
| **Second line therapy for metastatic** | | | |
| LHRHa + abiraterone | 42% | LHRHa (e.g. leuprorelin 11.25mg every 3 months), with abiraterone 1000mg until cancer progression | Survey of local experts |
| LHRHa + docetaxel | 58% | LHRHa (e.g. leuprorelin 11.25mg every 3 months), docetaxel 75mg per square meter of body surface area every 3 weeks for 6 cycles |  |
| **Care for castration-resistant prostate cancer** | | | |
| Abiraterone | 36% | Abiraterone 1000mg until cancer progression | Survey of local experts |
| Enzalutamide | 23% | Enzalutamide 160mg until cancer progression |  |
| Radium-223 | 1% | Monthly injection for 6 cycles |  |
| Docetaxel | 31% | Docetaxel 75mg per square meter of body surface area every 3 weeks for 10 cycles |  |
| Docetaxel + cabazitaxel | 2% | Docetaxel with cabazitaxel 20mg per square meter of body surface area every 3 weeks for 10 cycles |  |
| Docetaxel + abiraterone | 7% | Docetaxel 75mg per square meter of body surface area every 3 weeks for 10 cycles  + abiraterone 1000mg until cancer progression |  |
| Docetaxel + radium-223 | 1% | Docetaxel 75mg per square meter of body surface area every 3 weeks for 10 cycles  + monthly injection for 6 cycles |  |
| **Palliative care** | | | |
| Palliative radiotherapy | 17% | 5 to 20 treatment sessions | Survey of local experts |
| Analgesics | 56 % | Morphine 90mg per day |  |
| Palliative androgen deprivation therapy | 61% | - |  |

**Abbreviation**: LHRHa, luteinizing hormone-releasing hormone agonist

**Notes**:

1. As patients can concurrently receive more than one type of palliative care, the total may not add up to 100%.
