# Supplementary material for: Cost-effectiveness of MRI targeted biopsy strategies for diagnosing prostate cancer in Singapore
Source: BMC Health Serv Res. 2021 Sep 3;21:909. doi: 10.1186/s12913-021-06916-0 (PMC8414680; doi:10.1186/s12913-021-06916-0)
Supplement: Supplementary file 3 — Additional file 3: Table S3. Percentage of the treatment-related complications. [file 12913_2021_6916_MOESM3_ESM.docx]

**Table S-3. Percentage of the treatment-related complications**

| Treatment | Complications | Likelihood of complication | Source |
| --- | --- | --- | --- |
| Radical prostatectomy | Erectile dysfunction | 69% | Survey of local experts |
|  | Urinary urgency | 16% |  |
| Radiotherapy | Erectile dysfunction | 59% |  |
|  | Urinary urgency | 24% |  |
|  | Bowel problem | 16% |  |
| Androgen deprivation therapy | Erectile dysfunction | 100% |  |
