# Supplementary material for: Cost-effectiveness of MRI targeted biopsy strategies for diagnosing prostate cancer in Singapore
Source: BMC Health Serv Res. 2021 Sep 3;21:909. doi: 10.1186/s12913-021-06916-0 (PMC8414680; doi:10.1186/s12913-021-06916-0)
Supplement: Supplementary file 4 — Additional file 4: Table S4. Comparison of overall survival of men from published prostate cancer data and current study’s modelled population. [file 12913_2021_6916_MOESM4_ESM.docx]

Table S-4. Comparison of overall survival of men from published prostate cancer data and current study’s modelled population

| **Number of simulated years** | **Overall survival from published prostate cancer data** | **Overall survival predicted by current study’s modelled population of men with prostate cancer** |
| --- | --- | --- |
| 1 | 1 | 1 |
| 5 | 0.946 | 0.950 |
| 10 | 0.843 | 0.847 |
| 15 | 0.665 | 0.686 |
| 20 | - | 0.487 |

**Notes:**

1. Simulated year 1 in the model corresponds to 65 years of age.
2. 15-year overall survival rates from published prostate cancer data were from the PREDICT Prostate multivariate model (Thurtle et al, 2019). The model was developed using records of men diagnosed with non-metastatic prostate cancer between 2000 and 2010 in Eastern England from the United Kingdom National Cancer Registration and Analysis Service (N=10,089). It was then externally validated using a prostate cancer cohort in Singapore (N=2,546) diagnosed between 1990 and 2015 from a single institution, Singapore General Hospital. Published prostate cancer data from the PREDICT Prostate multivariate model were weighted by the proportion of low-, intermediate-, and high-risk localized prostate cancer.
