# Supplementary material for: Cost-effectiveness of MRI targeted biopsy strategies for diagnosing prostate cancer in Singapore
Source: BMC Health Serv Res. 2021 Sep 3;21:909. doi: 10.1186/s12913-021-06916-0 (PMC8414680; doi:10.1186/s12913-021-06916-0)
Supplement: Supplementary file 5 — Additional file 5: Table S5. Distribution of patients with localised prostate cancer across risk status. [file 12913_2021_6916_MOESM5_ESM.docx]

Table S-5. Distribution of patients with localised prostate cancer across risk status

| ISUP grade grouping | Risk status | Proportion of patients with localised prostate cancer with this risk status | Source |
| --- | --- | --- | --- |
| 1 | Low risk (PSA <10 ng/ml; Gleason score <7; and cT1 to 2a) | 31% | Survey of local experts |
| 2 to 3 | Intermediate risk (PSA 10 to 20 ng/ml; or Gleason score =7; or cT2b) | 44% |  |
| 4 to 5 | High risk (PSA >20 ng/ml; or Gleason score >7; or above cT2c) | 25% |  |

**Abbreviations:** cT, clinical cancer tumor (T) staging, ISUP, International Society of Urology Pathology; GS, Gleason score; PSA, prostate-specific antigen
