# Supplementary material for: Cost-effectiveness of MRI targeted biopsy strategies for diagnosing prostate cancer in Singapore
Source: BMC Health Serv Res. 2021 Sep 3;21:909. doi: 10.1186/s12913-021-06916-0 (PMC8414680; doi:10.1186/s12913-021-06916-0)
Supplement: Supplementary file 6 — Additional file 6: Table S6. Summary of one-way sensitivity analyses parameters and results - Strategy 4 vs Strategy 1. [file 12913_2021_6916_MOESM6_ESM.docx]

Table S-6. Summary of one-way sensitivity analyses parameters and results - Strategy 4 vs Strategy 1

| Model parameter | Parameter value (lower limit, upper limit) | ICER (cost in USD per QALY gained) | |
| --- | --- | --- | --- |
|  |  | Lower limit | Upper limit |
| **Base case** | | **19,175** | |
| Annual discounting rate | 0.03 (0.01; 0.05) | 15,755 | 23,022 |
| Prevalence of prostate cancer in men referred for first biopsy | 0.377 (0.34; 0.41) | 17,831 | 21,547 |
| Distribution of low-risk prostate cancer | 0.31 (0.27; 0.34) | 17,745 | 20,844 |
| Probability of detecting low-risk cancer given true low-risk cancer using systematic biopsy as first biopsy | 0.35 (0.25; 0.45) | 17,913 | 20,764 |
| Probability of detecting clinically significant cancer given true intermediate-risk cancer using MRI targeted biopsy as first biopsy | 0.79 (0.71; 0.87) | 17,397 | 20,207 |
| Probability of low-risk cancer classified as suspicious clinically significant cancer by mpMRI | 0.56 (0.46; 0.67) | 17,480 | 20,256 |
| Probability of detecting low-risk given true intermediate-risk cancer using MRI targeted biopsy as first biopsy | 0.08 (0.06; 0.16) | 17,441 | 19,932 |
| Probability of no cancer classified as suspicious clinically significant cancer by mpMRI | 0.5 (0.43; 0.58) | 17,971 | 19,989 |
| Charge of systematic biopsy | 1,124 (1,012; 1,236) | 18,495 | 19,855 |
| Distribution of intermediate-risk prostate cancer | 0.44 (0.4; 0.49) | 18,896 | 19,837 |
| Probability of intermediate-risk cancer being classified as suspicious clinically significant cancer by mpMRI | 0.87 (0.83; 0.91) | 18,527 | 19,457 |
| Charge of saturation biopsy | 1,565 (1,409; 1,722) | 18,748 | 19,603 |
| Utility weight for no cancer | 0.85 (0.83; 0.86) | 18,940 | 19,663 |
| Probability of detecting low-risk cancer given true intermediate-risk cancer using systematic biopsy as the second biopsy | 0.1 (0.07; 0.13) | 18,833 | 19,304 |
| Utility weight for intermediate-risk localized prostate cancer | 0.81 (0.803; 0.817) | 19,001 | 19,352 |
| Charge of mpMRI | 827 (744; 910) | 19,042 | 19,309 |
| Probability of detecting clinically significant cancer given intermediate-risk cancer using systematic biopsy as second biopsy | 0.35 (0.32; 0.38) | 18,927 | 19,175 |
| Charge of urology visit | 82 (73; 90) | 19,110 | 19,241 |
| Utility weight for low-risk localized prostate cancer | 0.84 (0.836; 0.844) | 19,122 | 19,229 |
| Utility decrements due to saturation biopsy | 0.007 (0.006; 0.008) | 19,123 | 19,224 |
| Probability of detecting low-risk given true low-risk cancer using systematic biopsy as the second biopsy | 0.45 (0.43; 0.47) | 19,148 | 19,244 |
| Utility decrements due to systematic biopsy | 0.004 (0.003; 0.004) | 19,130 | 19,220 |
| Charge of PSA test | 41 (37; 45) | 19,144 | 19,206 |
| Utility weight of patients with metastatic disease | 0.67 (0.660; 0.680) | 19,151 | 19,199 |
| Charge of MRI-US fusion targeted biopsy platform | 288 (259; 317) | 19,173 | 19,178 |
| Utility weight of patients with high-risk localized prostate cancer | 0.71 (0.701; 0.719) | 19,175 | 19,175 |

**Abbreviations**: ICER, incremental cost-effectiveness ratio; MRI, magnetic resonance imaging; mpMRI, multi-parametric magnetic resonance imaging; PSA, prostate-specific antigen; USD, US dollar

**Notes**:

1. MRI targeted biopsy refers to the administration of MRI targeted biopsy combined with systematic biopsy following positive mpMRI.
2. Parameters that are highlighted indicate that the upper bound limit of the parameter led to a lower ICER; parameters that are not highlighted indicate that the upper bound limit of the parameter led to higher ICER.
