# Supplementary material for: Cost-effectiveness of MRI targeted biopsy strategies for diagnosing prostate cancer in Singapore
Source: BMC Health Serv Res. 2021 Sep 3;21:909. doi: 10.1186/s12913-021-06916-0 (PMC8414680; doi:10.1186/s12913-021-06916-0)
Supplement: Supplementary file 7 — Additional file 7: Fig. S1. ICER tornado diagram for Strategy 4 vs Strategy 1 (full chart). [file 12913_2021_6916_MOESM7_ESM.docx]

**Figure S-1. ICER tornado diagram for Strategy 4 vs Strategy 1 (full chart)**


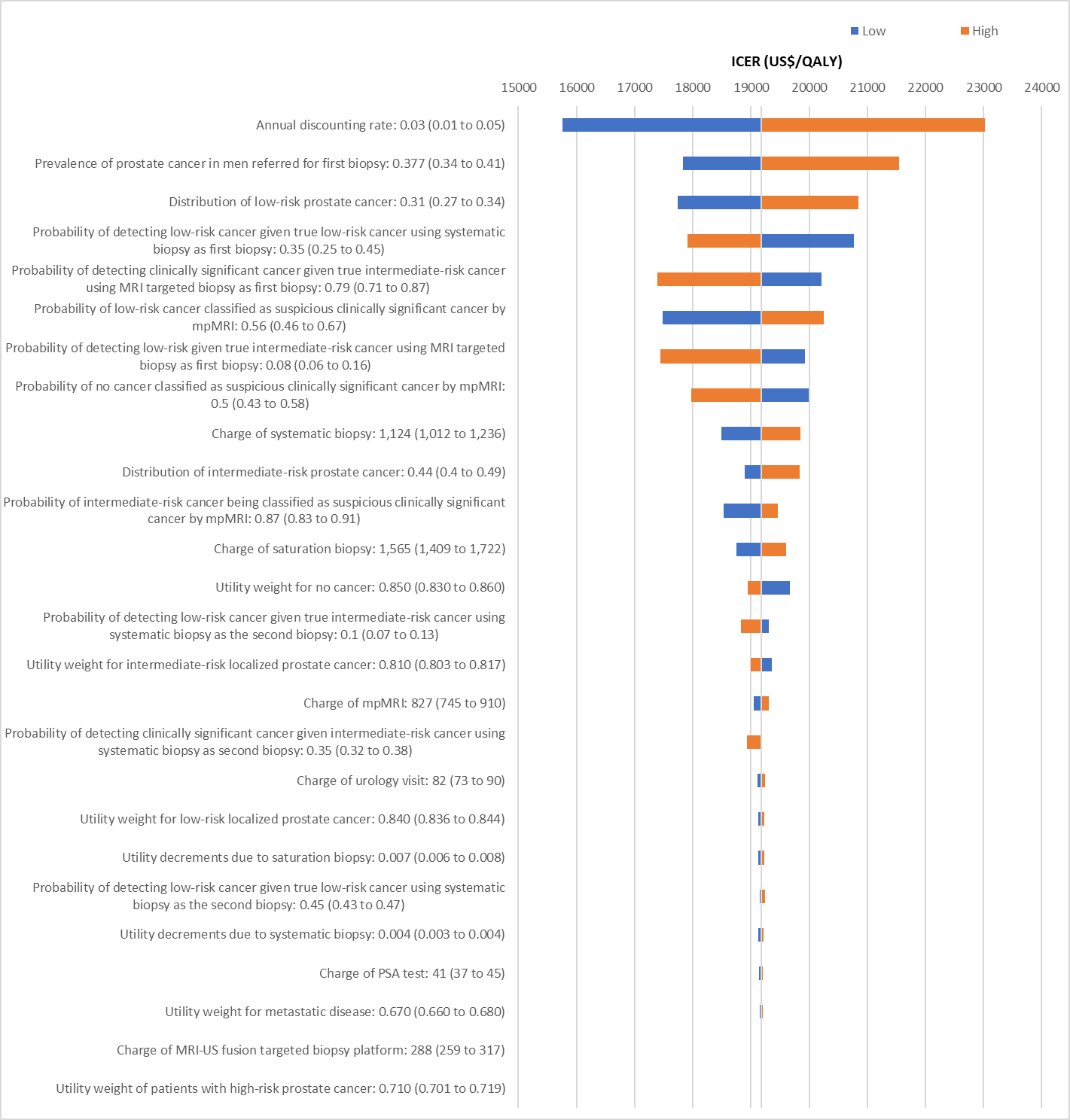


**Abbreviations**: ICER, incremental cost-effectiveness ratio; MRI, magnetic resonance imaging; mpMRI, multi-parametric magnetic resonance imaging; PSA, prostate-specific antigen

**Notes:**

1. Strategy 1: MRI targeted biopsy

Strategy 4: MRI targeted biopsy 🡪 Systematic biopsy 🡪 Saturation biopsy

1. MRI targeted biopsy refers to the administration of MRI targeted biopsy combined with systematic biopsy following a positive mpMRI.
2. Blue bars denote the ICERs when the parameter’s lower bound limit is tested; red bars denote the ICERs when the parameter’s upper bound limit is tested.
