# Supplementary material for: Cost-effectiveness of MRI targeted biopsy strategies for diagnosing prostate cancer in Singapore
Source: BMC Health Serv Res. 2021 Sep 3;21:909. doi: 10.1186/s12913-021-06916-0 (PMC8414680; doi:10.1186/s12913-021-06916-0)
Supplement: Supplementary file 8 — Additional file 8: Table S7. Probabilistic sensitivity analyses of all strategies for base case. [file 12913_2021_6916_MOESM8_ESM.docx]

Table S-7. Probabilistic sensitivity analyses of all strategies for base case

| Diagnostic strategy | Comparator | Costs (USD) | Incremental costs | Effectiveness (QALYs) | Incremental effectiveness | ICER (cost in USD per QALY gained) |
| --- | --- | --- | --- | --- | --- | --- |
| Strategy 1: MRI targeted biopsy | - | 9,761 | - | 10.2218 | - | - |
| Strategy 2: MRI targeted biopsy 🡪 Systematic biopsy | Strategy 1 | 10,124 | 363 | 10.2445 | 0.0227 | 15,990 |
| Strategy 3: MRI targeted biopsy 🡪 Saturation biopsy | Strategy 2 | 10,414 | 290 | 10.24 | -0.0045 | Dominated by Strategy 2 |
| Strategy 4: MRI targeted biopsy 🡪 Systematic biopsy 🡪 Saturation biopsy | Strategy 2 | 10,420 | 295 | 10.258 | 0.0135 | 21,826 |
| Strategy 5: Systematic biopsy 🡪 MRI targeted biopsy | Strategy 4 | 10,821 | 402 | 10.2549 | -0.0031 | Dominated by Strategy 4 |
| Strategy 6: Systematic biopsy 🡪 MRI targeted biopsy 🡪 Saturation biopsy | Strategy 4 | 11,045 | 625 | 10.2688 | 0.0108 | 58,097 |

**Abbreviations**: ICER, incremental cost-effectiveness ratio; mpMRI, multi-parametric magnetic resonance imaging; QALY, quality adjusted life year; USD, US dollar

**Notes**:

1. MRI targeted biopsy refers to the administration of MRI targeted biopsy combined with systematic biopsy following a positive mpMRI.
2. Arrow (🡪) refers to next sequence of diagnostic test following a negative biopsy result
3. The diagnostic strategies are organized from the least costly to most mostly strategy. The incremental cost and effectiveness of each strategy is calculated by comparing against the preceding strategy that is not dominated. A dominated strategy is more costly and less effective than the strategy in the immediately preceding row. The strategies highlighted in blue are not dominated; those not highlighted are dominated.
